# Antioxidant, Gastroprotective, Cytotoxic Activities and UHPLC PDA-Q Orbitrap Mass Spectrometry Identification of Metabolites in *Baccharis grisebachii* Decoction

**DOI:** 10.3390/molecules24061085

**Published:** 2019-03-19

**Authors:** Jessica Gómez, Mario J. Simirgiotis, Beatriz Lima, Jésica D. Paredes, Carlos M. Villegas Gabutti, Carlos Gamarra-Luques, Jorge Bórquez, Lorena Luna, Graciela H. Wendel, Alejandra O. Maria, Gabriela E. Feresin, Alejandro Tapia

**Affiliations:** 1 Instituto de Biotecnología-Instituto de Ciencias Básicas, Universidad Nacional de San Juan, Av. Libertador General San Martín 1109 (O), San Juan CP 5400, Argentina; jesicagomez674@gmail.com (J.G.); blima@unsj.edu.ar (B.L.); lluna@unsj.edu.ar (L.L.); gferesin@unsj.edu.ar (G.E.F.); 2 CONICET (Consejo Nacional de Ciencia y Tecnología), CABA, Buenos Aires C1405DJR, Argentina; cgamarraluques@gmail.com; 3 Instituto de Farmacia, Facultad de Ciencias, Universidad Austral de Chile, Campus Isla Teja, Valdivia 5090000, Chile; 4 Center for Interdisciplinary Studies on the Nervous System (CISNe), Universidad Austral de Chile, Valdivia 5090000, Chile; 5 Farmacología, Facultad de Química, Bioquímica y Farmacia, Universidad Nacional de San Luis, Chacabuco y Pedernera, San Luis CP5700, Argentina; jdparedes@unsl.edu.ar (J.D.P.); cmville@gmail.com (C.M.V.G.); gwendel@unsl.edu.ar (G.H.W.); alejandraomaria@gmail.com (A.O.M.); 6 Instituto de Medicina y Biología Experimental de Cuyo (IMBECU), CCT-Mendoza CONICET, Mendoza CP5500, Argentina; 7 Facultad de Ciencias Médicas, Universidad Nacional de Cuyo, Mendoza CP5500, Argentina; 8 Laboratorio de Productos Naturales Depto. de Química, Facultad de Ciencias, Universidad de Antofagasta, Av. Coloso S-N, Antofagasta 1240000, Chile; jorge.borquez@uantof.cl

**Keywords:** Argentinean plants, UHPLC Orbitrap (Q-OT), flavonoids, cinnamic acids derivatives

## Abstract

The decoction of the local plant *Baccharis grisebachii* is used as a digestive, gastroprotective, external cicatrizing agent and antiseptic in Argentine. A lyophilized decoction (BLD) from the aerial parts of this plant was evaluated regarding its anti-ulcer, antioxidant and cytotoxic activities and the bioactivities were supported by UHPLC-MS metabolome fingerprinting which revealed the presence of several small bioactive compounds. The antioxidant properties were evaluated by DPPH, TEAC, FRAP and lipoperoxidation inhibition in erythrocytes methods, and the antibacterial activity was evaluated according to the Clinical and Laboratory Standards Institute (CLSI) guidelines. The BLD showed a moderate free radical scavenging activity in the DPPH (EC_50_ = 106 µg/mL) and lipid peroxidation in erythrocytes assays (67%, at 250 µg/mL). However, the BLD had the highest gastroprotective effect at a dose of 750 mg/kg with a ninety-three percent inhibition of damage through a mechanism that involve NO and prostaglandins using the ethanol-induced gastric damage in a standard rat model. On the other hand, BLD does not induce cytotoxic changes on human tumor and no-tumor cell lines at the concentrations assayed. Regarding the metabolomic analysis, thirty-one compounds were detected and 30 identified based on UHPLC-OT-MS including twelve flavonoids, eleven cinnamic acid derivatives, one coumarin, one stilbene and two other different phenolic compounds. The results support that the medicinal decoction of *Baccharis grisebachii* is a valuable natural product with gastroprotective effects and with potential to improve human health that opens a pathway for the development of important phytomedicine products.

## 1. Introduction

The endemic Andean species *Baccharis grisebachii* Hieron (Asteraceae, vernacular name ‘quilchamalí’) is used as a digestive, gastroprotective, external cicatrizing agent and antiseptic in Argentine [1]. This bush is one of the most demanded and commercialized species by the herbalists or natural products stores in the central western region of Argentina [2]. The chemistry and biological activity focused on the organic extracts from this plant have been previously reported [3,4,5,6]. The chemical analysis of the resinous exudate has allowed the characterization and isolation of diterpenes, flavones, *p*-coumaric acid derivatives and flavonoids, while antimicrobial and anti-oxidant properties have been also described [4,5,6].

The in vitro cytotoxic properties on human oral epidermis cancer cells of extracts obtained from the aerial parts of *B. grisebachii* as well as its capacity to reduce oxidative stress and to synthesize stress proteins have been also studied [7,8], while the antimicrobial activity and chemical composition of essential oils have been also reported [9]. Additionally, the anatomical characters of *B. grisebachii* for specific identification and quality control have also been reported [10].

Until now, there are no reports about chemical characterization and the biological activities of the decoction obtained from this species. On the other hand, the use of UHPLC coupled to hybrid state-of-the-art mass spectrometers, such as quadrupole Orbitrap (Q-OT), is becoming a key tool for the rapid detection, identification and characterization of medicinal plant metabolites. A considerable number of Andean species, mainly from Chile and Argentina, have been recently reported using this technology [11,12,13,14,15,16].

The main goals and novelty of this work are the gastroprotective, antioxidant and antibacterial effects plus cytotoxicity on models of tumoral and non-tumoral human cell lines, complemented with the full metabolome polyphenolic profile using a hybrid high-resolution mass spectrometer of the lyophilized decoction (BLD) from the medicinal plant *B. grisebachii*, to support the reputed properties for the treatment of digestive ailments and other reported medicinal properties of this plant.

## 2. Results and Discussion

### 2.1. Total Phenolic and Flavonoids Contents, Antioxidant and Antimicrobial Activities

The BLD from aerial parts from *B. grisebachii* was assessed in vitro for total content of phenolics and flavonoids in addition to antioxidant properties (Table 1). The BLD showed a content of phenolic compounds of 67 mg GAE/g BLD, five percent of them corresponds to flavonoids (5.3 mg QE/g BLD). Reactive oxygen species (ROS) are derived of the many sources, including mitochondria, xanthine oxidase, uncoupled nitric oxide synthases and NADPH oxidase [17,18]. Oxidative stress is mainly caused by ROS damage normal organs, leading to a gradual loss of vital physiological function *B. grisebachii* BLD displayed a free radical scavenging activity in the DPPH and lipid peroxidation of the erythrocytes assays, while no significant effect in the FRAP and ABTS antioxidant assays were found (Table 1). The DPPH assay is widely used for quickly assessing the ability of polyphenols to transfer labile H atoms to radicals in methanol solution, which is likely the mechanism of antioxidant protection [19]. This effect could be related the presence of hydrogen-donating compounds, which are probably present in the polar decoction. The antioxidant capacity detected is in concordance with the content of total phenolics in BLD.

On the other hand, in a cell-based model including human erythrocytes, lipid peroxidation was studied to evaluate the biological relevance of the antioxidant capacity of the decoction. The results showed that BLD prevented the hemolytic effect of the rupture of cell membranes induced by lipid peroxidation (67%, at 250 µg/mL). This value showed resemblance to that evidenced by the reference compound catechin that showed an inhibition of the lipoperoxidation of 72% at 100 µg/mL.

In literature, several reports showed the antioxidant capacity of plant in the genus *Baccharis*. The free radical scavenger capacity of the DPPH radical of *B. trimera* aqueous extract (IC_50_ values of 415 μg/mL) was reported [20,21]. Moreover, the antioxidant activity of the essential oil of *B. trinervis* through capture of the DPPH radical, and the model system of oxidation of β-carotene/linoleic acid were evaluated, obtaining IC_50_ values of 49.0 mg/mL and 28.87 mg/mL, respectively [22]. Recently, the antioxidant effect of the exudate from *B. tola* by testing the reducing power of the ferric ion (0.05 mM ET/g dry plant), and DPPH assay (IC_50_ = 9.24 ± 0.23 μg/mL) were reported [23]. Regarding *B. grisebachii*, the free radical scavengers and lipoperoxidation inhibition in erythrocytes of several extracts, namely hexane, dichloromethane and methanol and bio-guided isolation of the main active *p*-coumaric acid derivatives and six aglycone flavonoids were reported [6].

Regarding the antibacterial activity, BLD was assayed against the pathogenic bacteria Gram-negative strains (ATCC and clinical isolates of *E. coli*), and Gram-positive *Staphylococcus aureus* strains methicillin sensitive (MSSA) and methicillin resistant (MRSA), and *S. aureus* coagulase negative-502 and *Streptococcus pyogenes*-1. The BLD did not exhibit relevant antimicrobial activities against the bacteria assayed, the MIC values were > 2000 µg/mL (data not shown).

### 2.2. Gastroprotective Effect induced by BLD

*B. grisebachii* lyophilized decoction (BLD) was tested in a model of ethanol induced acute gastric lesion in rats. The oral treatment of animals with BLD at 250, 500 and 750 mg/kg doses reduced the gastric lesions in a dose-dependent manner.

**Figure 1:**
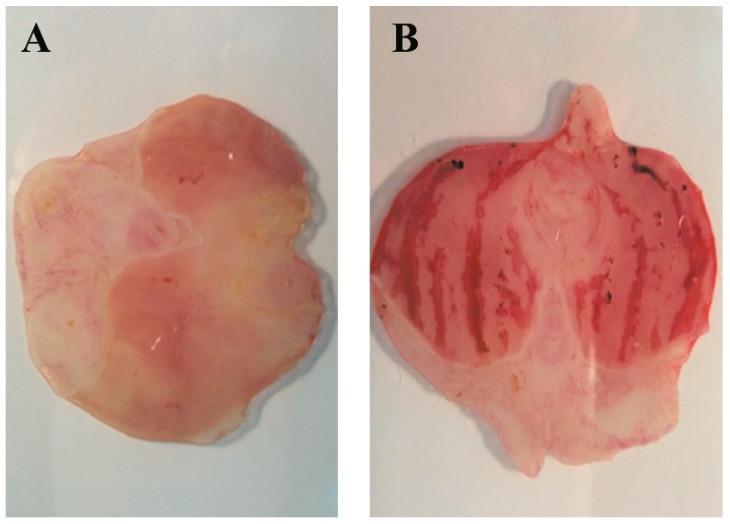
Effects of BLD at 750 mg/kg (**A**) on the macroscopic aspect of stomach in ethanol-induced gastric lesions in rats (**B**).

The results indicated that the dose of 750 mg of BLD/kg showed the highest significant cytoprotective effect (93% inhibition of damage) (Table 2, Figure 1). Also, the dose of 500 mg of BLD/kg showed a meaningful antiulcer effect of the 56%; while no protection was observed in the treatment with the dose of 250 mg/kg. Meanwhile omeprazole, the reference anti-ulcer drug, administered at 60 mg/kg showed 34% inhibition of ethanol induced damage.

Several authors have reported the cytoprotective capacity of the genus *Baccharis*. Gonzales et al. reported the cytoprotective activity of *B. genistelloides* and *B. rubricaulis* extracts administered orally (*n* = 6) with inhibition values of 85.7 and 64.3% at 1250 mg/kg, respectively [24]. Likewise, Baggio et al. reported the effect of *B. illinita* aqueous and hydro-alcoholic extracts administered orally (*n* = 6) with a moderated decrease in gastric lesions (50% at 1000 mg/kg) [25].

Vidari et al. evaluated the antiulcer and antidiarrheal effects in vivo of *B. teindalensis* ethanol extracts administered orally (*n* = 8–10) [26] while Lemos et al. reported the anti-ulcer property of the *B. dracunculifolia* hydro-alcoholic extract administered orally (five groups, *n* = 6) at a dose of 50, 250 and 500 mg/kg, showing a decrease in the total ulcer area of 79.9, 92.7 and 95%, respectively [27]. Likewise, other authors evaluated the *B. dracunculifolia* essential oil administered orally (five groups. *n* = 6) obtaining, for doses of 50, 250 and 500 mg/kg, an ulcer inhibition of 42.79, 45.70 and 61.61%, respectively [28]. Moreover, the lyophilized extract of *B. trimera* showed a reduction of 90% of the lesion area at a dose of 400 mg/kg [29]. On the other hand, the cytoprotective effect of the hydroethanolic extract of this species was evaluated in two models of gastric lesions: induced by ethanol and acetic acid; which showed a significant reduction in the area of the lesion and oxidative stress induced by the consumption of necrotizing agents [30].

Moreover, the possible involvement of NO and prostaglandins in the mechanism of action of the decoction in the gastroprotective model was also assessed. Since vascular changes in gastric mucosa appear to be the most pronounced feature of absolute ethanol induced injury, maintenance of mucosal vasculature and normal blood flow may be the major mechanism of cytoprotection. It has been demonstrated that the gastric mucosa produces endogenous NO derived from l-arginine, and that NO participates in gastric defense mechanisms by regulating the gastric mucosal blood flow [31].

Intraperitoneal treatment of rats with a non-selective inhibitor of NO synthase, Nω-nitro-l-arginine (L-NNA, 70 mg/kg) was able to reverse the gastroprotective effect caused by BLD (Ulcer index: 3 ± 0.4; *p* < 0.001 vs. BLD 750 mg/kg + EtOH group). This result suggests that endogenous NO partly participate in the protective effect of BLD.

Regarding exogenous prostaglandins, it was reported that these compounds protect the gastric mucosa against necrotizing agents, while mild irritants protect the gastric mucosa against damage via induction of endogenous prostaglandins as well [32,33]. The protective action of gut hormones has been attributed to the release of prostaglandins because it could be abolished by the pretreatment with indomethacin and restored by the addition of exogenous PGE2 [34]. Pre-treatment with a non-selective inhibitor of cyclooxygenase (indomethacin, 10 mg/kg, i.p.) significantly attenuated the BLD gastroprotection (Ulcer index: 2.16 ± 0.3; *p* < 0.001 vs. BLD 750 mg/kg + EtOH group), suggesting a role of endogenous prostaglandins in BLD gastroprotection.

### 2.3. Toxicity Study of B. grisebachii Lyophilized Decoction on Human Cell Lines

**Figure 2:**
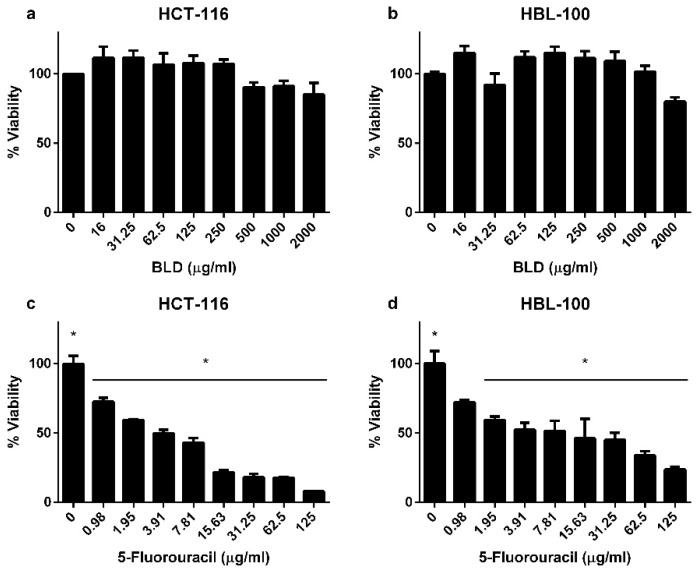
MTT viability of human colorectal cancer and epithelial non-tumoral mammary cell lines (HCT-116 and HBL-100, respectively) in a dose-response experimental design for 72 h. When BLD was used on both cell lines (**a**,**b**), the obtained results showed no-significant changes in cell viability among treatments and control groups. However, statistical differences were found in the treatment with 5-fluorouracil from 0.98 μg/mL in HCT-116 (**c**) and 1.95 μg mL in HBL-100 (**d**). ANOVA followed by Dunnett’s comparison test was used (asterisk indicates statistical significance, *p* ≤ 0.05).

The cytotoxic activity of the decoction was tested using the MTT assay, by the dose-response experimental design in human tumoral (HCT-116) and non-tumoral (HBL-100) cell lines (Figure 2). After 72 h of treatment exposure at the indicated doses, the cells viability evidenced no significant differences among control (0 µg/mL) and the treated groups (range 16–2000 µg/mL). However, when 5-fluorouracil was used as positive control compound, the treatment evidenced cytotoxicity in both cell lines, while the treatment of HCT-116 cells showed significant differences at doses from 0.98 µg/mL; in the HBL-100 non-tumoral cells, significant cytoxicity resulted at doses starting from 1.95 μg/mL. In accordance to this, it is possible to support the treatment with *B. grisebachii* as a non-cytotoxic treatment.

### 2.4. UHPLC-OT Analysis of BLD

**Figure 3:**
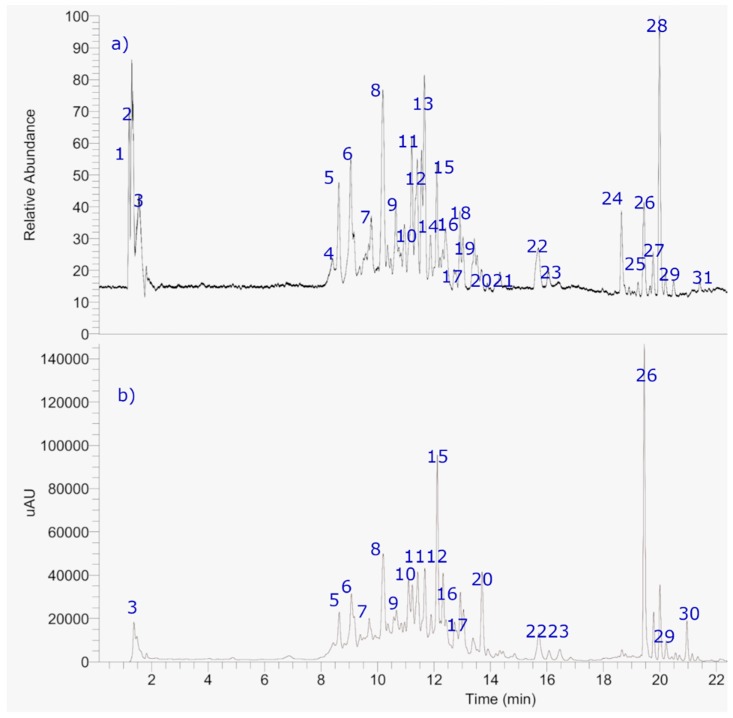
The HPLC-MS Fingerprints of *Baccharis grisebachii* lyophilized decoction: (**a**) The total Ion Current (TIC) chromatogram and (**b**) the UV-vis chromatogram at 280 nm.

The use of HPLC or UHPLC coupled to hybrid state-of-the-art mass spectrometers, such as quadrupole-time of flight (Q-ToF) quadrupole-Orbitrap^®^ (Q-OT), or ion cyclotron (FTIC) are becoming a key tool for the rapid and accurate analysis of phenolic substances in organic samples. For the first time, thirty-one major compounds were detected and identified based on the UHPLC OT-MS and PDA analysis on the decoction (BLD) of *B. grisebachii* (Figure 3, Figure S1, Supplementary Material, and Table 3). From them, twelve (peaks 9, 10, 12, 13, 17, 22–26, 29 and 30) correspond to flavonoids and eleven to cinnamic derivatives (peaks 5–8, 11, 14, 18-20, 27 and 28), one to a coumarin (peak 16) and two to other different phenolic compounds (peaks 4, and 31) and one stilbene (peak 15). Figure S1 (Supplementary Material) shows the full HR-MS spectra and structures of some of the representative substances. The metabolomics identification is explained below in detail.

• Flavonoids

Several compounds were methyl derivatives of simple flavonoids, which is consistent with the chemistry of plants from arid environments. Peaks 9 and 17 were identified as the simple flavonoids quercetin and kaempferol. Quercetin is the most abundant antioxidant flavonoid utilized as a nutritional supplement and as a phytochemical remedy for a variety of diseases like diabetes/obesity and circulatory dysfunction, including inflammation as well as mood disorders. Its chemical structure support their strong antioxidant activity, which potentially enables it to quench free radicals from forming resonance-stabilized phenoxyl radicals [35].

Additionally, quercetin has cytoprotective effects and it stimulates gastric epithelial proliferation, so regarded as a valuable therapeutic agent for colitis and gastric ulcer. Quercetin is also very effective for the healing of common mouth ulcers [36].

Regarding kaempferol, peak 17, its anti-oxidant/anti-inflammatory effects have been demonstrated in various disease models, including those for encephalomyelitis, diabetes, asthma, and carcinogenesis. Moreover, kaempferol act as a scavenger of free radicals and superoxide radicals as well as preserve the activity of various anti-oxidant enzymes such as catalase, glutathione peroxidase, and glutathione-S-transferase [37]. At the molecular level, kaempferol has been reported to modulate a number of key elements in cellular signal transduction pathways linked to apoptosis, angiogenesis, inflammation, and metastasis [38]. Interestingly, kaempferol was found to reduce the β-sheet content augmenting the mutant conformational stability and flexibility relative to that of kaempferide, peak 25, the methylated derivative, in amyotropic lateral sclerosis (kaempferide, CAS 491-54-3, PubChem CID 5281666) [39].

Peak 12 was identified as rutin (C_27_H_29_O_16_) and peak 13 has been characterized as the methylated quercetin derivative isorhamnetin (C_16_H_11_O_7_), while peak 10 showing a parent ion at *m*/*z*: 447.09088 and a daughter ion at *m*/*z*: 285.03894 (kaempferol) as a kaempferol hexoside, possibly kaempferol 3-*O*-glucoside or kaempferol 3-*O*-galactoside, the first one has been regarded as antiaging compound [40] and affects the endothelial function of *Ginkgo biloba* extract [41] and the second prevented carbon tetrachloride-induced liver injury in mice, being regarded as a liver protective compound [42]. Peak 24 was tentatively identified as the demethylated rhamnacin (3,7-dimethyl quercetin, C_17_H_13_O_7_), and peak 29 as nevadesin (5,7-dihydroxy-6,8,4′-trimethoxyflavone) which has a variety of pharmacological effects such as anti-mycobacterium tuberculosis, antitussive, anti-inflammatory, antihypertensive and free radical-scavenging activities effects [43,44,45]. Likewise, peaks 22 and 23 were identified as dimethyl myricetin derivatives one of them probably syringetin [46] (demethylated molecules at 330.03610, (demethylated molecule) 315.01309 (didemethylated molecule). Peak 26 was determined as a polymethoxylated flavonol possibly jaceidin (C_18_H_15_O_8_, CAS 19536-25-5, PubChem CID 5464461) [47] which has demonstrated protective activity against chromosomal damage in mitogen induced human lymphocytes [48] and peak 30 as a polymethoxylated flavonol possibly, the methyl jaceidin derivative casticin, [49] this compound is very bioactive compound and potent anti-inflammatory agent [50,51,52].

• Coumarins

Peak 16 was identified as the coumarin (UV max around 300 nm) fraxetin (C_10_H_8_O_5_) [53].

• Stilbenes

Peak 15 was tentatively identified as the stilbene glucoside: rhapontin (parent ion at *m*/*z*: 419.13287, C_21_H_23_O_9^−^_) producing a daughter ion at *m*/*z*: 257.08051 (C_15_H_13_O_4_, pontigenin). This compound has been reported with anti-diabetic anti-allergic and antithrombotic activities [54].

• Hydroxycinnamic acids

Several compounds were identified as hydroxycinnamic acid derivatives (UV max around 300 nm). Peak 5, with [M − H]^−^ ions at *m*/*z*: 353.08671 caffeoylquinic acid (C_16_H_17_O_9^−^_) [55] and peak 7 as p-coumaroyl-quinic acid (C_16_H_17_O_8^−^_) [56]. Besides, isomer compounds detected with peaks 6, 8 and 18 with [M − H]^−^ ions at *m*/*z*: 367.10162, 367.10159 and 367.10162 were identified as isomers of feruloyl-quinic acids (C_17_H_19_O_9^−^_) [55,57,58] and peak 11 with [M − H]^−^ ions at *m*/*z*: 265.10705 as 2(3-hydroxy-isopentyl) caffeic acid (C_14_H_17_O_5^−^_) and finally, peak 14 was identified as caffeoylquinic acid (C_16_H_17_O_9^−^_) [55].

Oxidative damage is considered a major mechanism in the pathogenesis of ulcer. Several phenolic acids such as caffeic, p-coumaric, ferulic and cinnamic acids have been documented to possess gastroprotective activity [59,60]. Furthermore, peak 19 was identified as 3-prenyl-4-hydroxycinnamic acid (3-prenyl-*p*-coumaric acid = drupanin) (C_14_H_15_O_3^−^_) with a daughter ion at *m*/*z*: 163.04005 (deprenylated molecule) which was isolated from the same source by some of us, and proved to be antioxidant and antimicrobial; the identity was verified by co-spiking with an authentic sample, and peak 20, 27 and 28 as its isomers, 2-prenyl-4-hydroxycinnamic acid (2-prenyl-*p*-coumaric acid), 3-prenyl-5-hydroxycinnamic acid and 2-prenyl-5-hydroxycinnamic acid.

• Other compounds

Peaks 2–4 were identified as gluconic acid (C_6_H_12_O_7_), identified by spiking experiments with authentic standards, quinic acid (C_7_H_12_O_6^−^_) [61] and hydroxybenzoic acid hexoside (C_13_H_15_O_8^−^_,) a compound common in plants [62], peak 21 as the cytoprotective dykellic acid (C_14_H_15_O_4_) [63] and peak 31 with a deprotonated molecule at *m*/*z*: 293.17380, matched the anticancer compound gingerol (C_17_H_25_O_4^−^_) [64], but since this compound is exclusive of ginger family plants respectively, this peak identification remains unknown (it could be also possibly botryenalol (C_17_H_25_O_4_, PubChem ID 15786208).

## 3. Materials and Methods

### 3.1. General Experimental Procedures

Ultra-pure water (<5 µg/L TOC) was obtained from a water purification system Arium 126 61316-RO, plus an Arium 611 UV unit (Sartorius, Goettingen, Germany). Methanol (HPLC grade) and formic acid (puriss. p.a. for mass spectrometry) from J. T. Baker (Phillipsburg, NJ, USA) were obtained. Commercial Folin-Ciocalteu (FC) reagent, 2,2-diphenyl-1-picrylhydrazyl (DPPH), ferric chloride hexahydrate, 2,4,6-tris(2-pyridyl)-s-triazine, trolox, quercetin, rutin, gluconic acid, kaempferol, isorhamnetin, chlorogenic acid, gallic acid, chloroform and DMSO were purchased from Sigma-Aldrich Chem. Co. (St Louis, MO, USA). Cefotaxime was from Argentia^®^ (Bristol-Myers Squibb, Buenos Aires, Argentina). Mueller–Hinton broth was provided by Laboratorio Britania (Buenos Aires, Argentina) Clarithromycin and metronidazole were purchased from Abbott Laboratories (Buenos Aires, Argentina), and Sigma-Aldrich, respectively. All other chemicals used were of reagent grade and obtained from the local market.

Identification of phenolic compounds by UHPLC-Q-OT-HESI-MS/MS. A Thermo Scientific Dionex Ultimate 3000 UHPLC system controlled by Chromeleon 7.2 Software (Thermo Fisher Scientific, Waltham, MA, USA) hyphenated with a high-resolution Q Exactive focus mass spectrometer (Bruker Daltonics, Bremen, Germany) were used for analysis. Nitrogen (purity >99.999%) obtained from a Zefiro nitrogen generator (Clantecnologica, Sevilla, Spain) was employed as both the collision and damping gas. All calibration and equipment parameters were set as previously reported [15]. LC parameters: The column used was a UHPLC C18 column (Acclaim, 150 mm × 4.6 mm ID, 5 µm, Restek Corporation, Bellefonte, PA, USA) operated at 25 °C. The detection was set at 254, 280, 320 and 440 nm, and PDA from 200 to 800 nm was recorded. Mobile phases were water/1% formic acid (A) and acetonitrile with 1% formic aqueous solution (B). The gradient program time (min), (% B) was: (0.00, 5); (5.00, 5); (10.00, 30); (15.00, 30); (20.00, 70); (25.00, 70); (35.00, 5) and 12 min for column equilibration. The flow rate was set at 1.00 mL min^−1^, and the injection volume: 10 µL. Standards and extracts dissolved in methanol were kept at 10 °C in the auto sampler. MS parameters: The HESI II and other parameters for the Q-orbitrap instrument were optimized also as previously reported [65].

### 3.2. Plant Material

*Baccharis grisebachii* was collected in San Juan Province, Argentina, during the flowering time on December 2016. The plant was identified by Dr M. Hadad, CIGEOBIO-CONICET, Universidad Nacional de San Juan, Argentina, and a voucher specimen has been previously deposited at the herbarium of the Escuela de Química y Farmacia, Universidad de Chile (SQF 21011), Santiago de Chile, Chile.

### 3.3. Lyophilized Decoction

The decoction was prepared at 10% weight/volume (500 g/5 L) of dried and milled plant using purified water by means of a PSA equipment. After 30 min of boiling, the decoction was filtered, cooled for 24 h in a freezer at −40 °C, and subsequently lyophilized in a LA-B3 RIFICOR equipment, obtaining a yield of 4 g of lyophilized decoction (BLD), each 100 mL of decoction (4% *w*/*v*). The BLD was stored in a freezer at −40 °C until its use in the different tests. The extraction procedure (BLD) was done three times.

### 3.4. Determination of Total Phenolics (TP) and Flavonoids (F) Content

The total phenolics (TP) and flavonoids (F) content of the extracts were determined by Folin-Ciocalteu and AlCl_3_ colorimetric methods, respectively [66]. The TP were expressed as milligrams of gallic acid equivalents (GAE) per gram of extracts (mg GAE/g extract). F were expressed as milligrams of quercetin equivalents (QE) per gram of extracts on (mg QE/g extracts). The values from triplicates were reported as the mean ± SD.

### 3.5. Antioxidant Activity

#### 3.5.1. DPPH Scavenging Activity

Free radical scavenger activity on DPPH free radical scavenging effects were assessed by the procedure previously described in References [6,65]. The scavenging activities were evaluated at 517 nm in a Multi-skan FC microplate photometer (Thermo Scientific). The analyses were performed in triplicate and values were reported as EC_50_ mean ± SD; being EC_50_, the extracts’ concentration provided 50% of radicals scavenging activity. Quercetin was used as a reference compound.

#### 3.5.2. Ferric-Reducing Antioxidant Power Assay (FRAP)

FRAP assay was performed in accordance to [66] with some modifications. Briefly, the FRAP solution was freshly prepared by mixing 10 mL of acetate buffer 300 mM at pH 3.6, 1 mL of ferric chloride hexahydrate 20 mM dissolved in distilled water and 1 mL of 2,4,6-tris(2-pyridyl)-*s*-triazine 10 mM dissolved in HCl 40 mM. Then, 10 µL of sample solution were mixed with 190 µL of the FRAP solution in 96-well microplates, in triplicate. Results were obtained by linear regression from a calibration plot obtained with Trolox (0–1 mmol/L). All samples were analyzed in triplicate. The results were expressed as mM TE/g extract.

#### 3.5.3. Trolox Equivalent Antioxidant Activity (TEAC) Assay

The TEAC assay was performed in accordance to Re et al., 1999 [67] with minor modifications. Briefly, 10 µL of the sample or Trolox standard was mixed with 200 µL of ABTS^•+^ (dissolved in PBS). The vortex was mixed for 10 s and the absorbance at 734 nm after a 4 min reaction at 30 °C was measured. The results were obtained by linear regression from a calibration plot constructed with Trolox (0–1 mmol L^−1^) and are expressed in TEAC values [68]. The TEAC value of samples is equivalent to the concentration of a Trolox solution. All samples were analyzed in triplicate. The results were expressed as mg TE/g extract.

#### 3.5.4. Lipid Peroxidation in Human Erythrocytes

The evaluation of lipid peroxidation in human erythrocytes was carried out as described by reference [65] with minor modifications. Human red blood cells obtained from healthy adult individuals were washed three times in cold phosphate buffered saline (PBS) by centrifugation at 3500 rpm. After washing, the cells were suspended in PBS, regulating the density to 1 mM hemoglobin in each reaction tube. The final cell suspension was incubated with different concentrations of the test compounds and dissolved in DMSO and PBS for 10 min at 37 °C. The final concentration of samples and controls in DMSO was 1%. After incubation, the cells were exposed to *tert*-butylhydroperoxide (1 mM/L) for 15 min at 37 °C under vigorous shaking. Then, the lipid peroxidation was determined indirectly by the TBARs formation. The results are expressed as a percentage of inhibition compared to the controls. Each determination was performed as a quadruplicate.

### 3.6. Toxicity Study of B. grisebachii Lyophilized Decoction on “in vitro” Human Cell Lines

#### 3.6.1. Cell Lines and Culture Conditions

Humans colorectal cancer cell line (HCT-116), and epithelial mammary non-tumoral cell line (HBL-100) were cultivated in DMEM media with 10% of fetal bovine serum, 100 IU of penicillin and 100 μg/mL streptomycin. Culture conditions were fixed at 37 °C, in a humidified atmosphere enriched by 5% CO_2_. Twenty-four h after cells seeding, the BLD was added to the culture media for 72 h. Both cell lines are commercially available from the American Type Culture Collection (ATCC, Manassas, VA, USA); catalog identification CCL-247 and HTB-124, respectively. There are not reported ethical considerations to consider when these cell lines are used for laboratory research purposes only.

#### 3.6.2. Cytotoxicity Assay by MTT

A colorimetric assay using MTT [3-(4,5-dimethylthiazol-2-yl)-2,5-diphenyltetrazolium bromide] was performed as was originally described by Mosmann, 1983 [69]. HCT-116 and HBL-100 cells were seeded in 96-well microplates (3–5 × 10^3^ cells/well/100 μL, respectively). In both cell lines, 24 h later, the medium was aspirated and replaced by the medium containing treatments. *B. grisebachii* BLD was used at concentrations ranging from 0 to 2.000 μg/mL; while, the chemotherapic 5-fluorouracil (Filaxis^®^, Buenos Aires, Argentina) was used as a control cytotoxic compound at concentrations ranging from 0 to 125 μg/mL.

After 72 h of treatment, the medium was replaced by 100 μL of MTT solution (0.5 mg/mL in DMEM, without phenol red or FBS); and cells were incubated for an additional 4 h. MTT solution was then removed and 100 μL of DMSO added; the plates were shaken for 10 min to dissolve the formazan crystals. The optical density was measured using a Thermo Scientific Multiscan microplate reader at 570 nm. The optical density obtained in untreated control cells was taken as 100% viability. Assays were performed three times in triplicate.

### 3.7. Induction of Gastric Lesions

#### 3.7.1. Animals

Male Wistar rats (200–250 g) were used. The animals, randomly assigned into groups (*n* = 6–8), were deprived of food for 24 h prior to starting the experiments and had free access to water.

#### 3.7.2. Induction of Gastric Lesions

Gastric lesions were produced according to the method of Robert et al., 1979. The experiments were carried out following Provision A.N.M.A.T. No. 6344/96, approved by the Institutional Committee for the Care and Use of Animals (CICUA), Protocol: F-284/17 UNSL, Argentine (Supplementary material). All rats were housed in wire mesh-bottomed cages throughout the study to prevent coprophagy. The necrotizing agent absolute ethanol was administered orally (p.o., 1 mL/animal), and 1 h later, the animals were euthanized by inhalation of carbon dioxide. The stomachs were removed, opened along the greater curvature and washed gently with ice-cold saline solution. The degree of erosion in the glandular part of the stomach was assessed from a scoring system designed by Marazzi-Uberti and Turba [70] from 0 (no erosions) to 5 (maximal damage). The results were expressed in terms of an Ulcer Index (IU) which is the average severity of erosions per rat for each group. The BLD concentrations were 250, 500 and 750 mg/kg and positive control omeprazole (60 mg/kg) or vehicle were administered orally 60 min prior to the necrotizing agent (p.o.). The involvement of prostaglandins and nitric oxide (NO) in the gastroprotection elicited by BLD was also evaluated. In another set of experiments, the animals were pretreated with a non-selective inhibitor of NO synthase, Nω-nitro-l-arginine (L-NNA, 70 mg/kg, i.p.) or a nonsteroidal anti-inflammatory drug which inhibits the enzyme cyclooxygenase (which synthesizes prostaglandins), indomethacin (10 mg/kg, i.p.). After 30 min, the rats received BLD (750 mg/kg) or vehicle (p.o.). Sixty minutes later, gastric damage was induced by administration of absolute ethanol (p.o.). The control groups received only vehicles or vehicles plus BLD. The animals were euthanized by inhalation of carbon dioxide after 1 h and Ulcer index was determined as described above.

### 3.8. Antibacterial Activity

The Microorganisms were: Gram-positive: *Staphylococcus aureus* methicillin-sensitive ATCC 29213, *Staphylococcusaureus* methicillin-resistant ATCC 43300, clinical isolates of *Staphylococcus coagulase* negative-502, *Streptococcus pyogenes*-1 (by Laboratorio de Microbiología, Hospital Marcial Quiroga, San Juan, Argentina); and Gram-negative: *Escherichia coli* ATCC 25922 and clinical isolates of *Escherichia coli* LM-2 (Laboratorio de Microbiología, Facultad de CienciasMédicas, Universidad Nacional de Cuyo, Mendoza, Argentina) were used.

An antibacterial susceptibility test in which the minimal inhibitory concentration (MIC) values were determined using the broth microdilution method according to the protocols of the Clinical and Laboratory Standards Institute [71]. The bacteria inoculum employed was 5 × 10^5^ CFU/mL. The stock solutions of extracts in the DMSO were prepared to give serial two-fold dilutions to obtain the final concentrations between 0.98 and 1000 µg/mL. Cefotaxime (Argentia^®^) was included in the assays as a positive control. The plates were incubated for 24 h at 37 °C. The activity was evaluated at 620 nm using a Multiskan FC instrument. The MIC values were defined as the lowest extract concentrations showing no bacterial growth after the incubation time. Tests were done in triplicates.

### 3.9. Statistical Analysis

Determinations of TP, TF, TA, DPPH, FRAP and TEAC were performed in triplicate and results are expressed as mean values ± SD. Results were analyzed by one-way ANOVA and significant differences between mean values were determined by Duncan’s test (*p* < 0.05). The statistical package InfoStat26 was used for statistical analyses. Statistical analysis data in toxicity assays are expressed as mean ± standard error (SEM). Data were analyzed using GraphPad Prism 5.0 software. ANOVA followed by Dunnett’s multiple comparison test was used to determine significant differences between groups. Statistical analysis in ulcerogenic assays was performed using GraphPad Prism version 5.00 for Windows and GraphPad InStat version 3.00 for Windows (GraphPad Software, San Diego, CA, USA, www.graphpad.com).

## 4. Conclusions

The findings in the present study indicate that *B. grisebachii* decoction (LD) displays a significant anti-ulcerogenic activity. The mode of action suggests that NO and prostaglandins possesses a potential role of in the gastroprotective effect. The identification for the first time of some small bioactive compounds in the aqueous extract carried out by UHPLC-MS studies, in addition to the free radical scavenging activity and the non-cytotoxic effects, partially supports the reputed properties of this plant for the treatment of digestive affections. Additionally, the global trend toward the use of natural aqueous preparations as pharmaceuticals rather than pure drugs opens a pathway for the development of a phytomedicinal product from *B. grisebachii* lyophilized decoction. More studies are needed to correlate the gastroprotective effects and the bioactive compounds in the plant.

## Figures and Tables

**Table 1:** The antioxidant, total phenolic and flavonoid content of *B. grisebachii* BLD.

Assay	Lyophilized Decoction (BLD)
Phenolic content	
Total phenolics (mg GAE/g extract)	62.46 ± 9.27
Flavonoids (mg QE/g extract)	5.30 ± 0.41
Antioxidant capacity	
DPPH (IC_50_ in µg/mL)	106.40 ± 22.48
FRAP (mM TE/g extract)	0.70 ± 0.19
TEAC (mg TE/g extract)	0.61 ± 0.04
Percentage ILP (at 250 µg/mL)	67.46 ± 1.05
Catechin (Percentage ILP at 100 µg/mL)	72.80± 3.32

**Table 2:** Gastroprotective effect of *B. grisebachii* LD on ethanol induced gastric lesions in rats.

*B. grisebachii* Treatment (mg/kg)	Gastroprotective Effect	
	Ulcer Index	% Lesion Reduction
LD (250)	4.4 ± 0.2	9.6
LD (500)	2.1 ± 0.5 ***	56.0
LD (750)	0.3 ± 0.2 ***	93.2
Omeprazole (60)	3.2 ± 0.2 **	34.2
Control EtOH	4.8 ± 0.1	0

Gastroprotective effect shown as mean Ulcer index ± error standard of mean (SEM) and percent lesion reduction compared with untreated controls. Omeprazole was used as reference drug. Asterisks denote significant differences from the control: *** *p* < 0.001 and ** *p* < 0.01 (ANOVA and posterior comparison by Tukey-Kramer).

**Table 3:** High-resolution UHPLC PDA-Q orbitrap identification of metabolites in *B. grisebachii* lyophilized decoction.

Peak #	Retention Time (min)	UV Max	Tentative Identification	Elemental Composition [M − H]^−^	Theoretical Mass (*m*/*z*)	Measured Mass (*m*/*z*)	Accuracy (δ ppm)	MS^n^ Ions
1	1.87	-	Unknown			272.95877	2.88	-
2	1.29	-	Gluconic acid *	C_6_H_12_O_7_^−^	195.04965	195.04993	−1.42	-
3	2.41	-	Quinic acid	C_7_H_12_O_6_^−^	191.05501	191.05478	1.23	-
4	8.38	310	Hydroxybenzoic acid hexoside	C_13_H_15_O_8_^−^	299.07614	299.07568	−1.53	137.02442
5	8.59	239–320	Caffeoylquinic acid (chlorogenic acid) *	C_16_H_17_O_9_^−^	353.08618	353.08671	2.31	191.05481 (quinic acid)
6	9.02	246–320	Feruloylquinic acid	C_17_H_19_O_9_^−^	367.10236	367.10162	−2.12	193.04915 (ferulic acid)
7	9.74	335	*p*-Coumaroylquinic acid	C_16_H_17_O_8_^−^	337.09137	337.09289	−1.25	163.0427 (coumaric moiety)
8	10.21	246–320	Feruloylquinic acid	C_17_H_19_O_9_^−^	367.10236	367.10159	−2.08	193.04915 (ferulic acid)
9	10.64	255–355	Quercetin *	C_15_H_10_O_7_^−^	301.03302	301.03428	−4.18	179.03343, 151.00220, 125.02163
10	10.93	265–365	Kaempferol hexoside	C_21_H_19_O_11_^−^	447.09219	447.09088	−2.9	285.03894 (kaempferol)
11	11.17	330	2 (3-Hydroxyisopentyl) caffeic acid	C_14_H_17_O_5_^−^	265.10675	265.10705	−1.13	191.04498, 179.03369, 173.04430
12	11.90	255–354	Rutin	C_27_H_29_O_16_^−^	609.14611	609.14532		301.03308, (quercetin) 271.02472
13	11.19	255–355	Isorhamnetin *	C_16_H_11_O_7_^−^	315.04956	315.04993	−1.16	300.02597 (demethylated molecule) 257.080051, 160.84082
14	11.38	246–335	Caffeoylquinic acid	C_16_H_17_O_9_^−^	353.08603	353.08671	−1.92	191.05481 (quinic acid)
15	11.56	270–312	Rapontin	C_21_H_23_O_9_^−^	419.13287	419.13366	−1.87	257.08051 (C_15_H_13_O_4_^−^ pontigenin) 213.09070, 173.04413
16	11.86	320–346	Fraxetin	C_10_H_8_O_5_^−^	207.02880	207.02852	−1.35	193.04932, 179.03372, 173.0443
17	12.21	265–365	Kaempferol *	C_15_H_9_O_6_^−^	285.03873	285.03936	−2.23	265.03394, 174.95479,160.84067, 151.00232,
18	12.43	330	Feruloylquinic acid	C_17_H_19_O_9_^−^	367.10236	367.10162	−2.08	193.04915 (ferulic acid)
19	12.89	335	3-Prenyl-4-hydroxycinnamic acid (3-prenyl-*p*-coumaric acid = drupanin) *	C_14_H_15_O_3_^−^	231.10130	231.10124	−1.15	187.11157, 163.04002 (deprenylated molecule)
20	12.94	335	2-Prenyl-4-hydroxycinnamic acid (2-Prenyl-*p*-coumaric acid)	C_14_H_15_O_3_^−^	231.10157	231.10124	−1.42	187.11157, 163.04005 (deprenylated molecule)
21	13.03	225	Dykellic acid	C_14_H_15_O_4_^−^	247.09649	247.09610	−1.56	
22	13.41	254–354	Dimethylmyricetin (syringetin)	C_17_H_13_O_8_^−^	345.05984	345.05991	−1.70	330.03610, (demethylated molecule) 315.01309 (di-demethylated molecule)
23	14.34	254–354	Dimethylmyricetin	C_17_H_13_O_8_^−^	345.06049	345.05991	−1.70	330.03625, (demethylated molecule) 315.01315 (di-demethylated molecule)
24	15.68	255–355	Rhamnacin (3,7-dimethyl quercetin)	C_17_H_13_O_7_^−^	329.06558	329.06506	−1.56	299.01822 (M-CH_3_) C_15_H_7_O_7_^−^
25	16.06	265–365	Kaempferide	C_16_H_11_O_6_^−^	299.05501	299.05463	−1.29	284.03119 (kaempferol)
26	18.63	254–330–354	Jaceidin	C_18_H_15_O_8_^−^	359.07559	359.07614	−1.53	344.05167, demethylated molecule 329.02853 di-demethylated molecule, 314.00159
27	19.24	335	Prenyl-5-hydroxycinnamic acid (3-prenyl-*m*-coumaric acid)	C_14_H_15_O_3_^−^	231.10118	231.10124	−1.38	187.11157
28	19.64	335	2-Prenyl-5-hydroxycinnamic acid (2-prenyl-*m*-coumaric acid)	C_14_H_15_O_3_^−^	231.10121	231.10124	−1.55	187.11157
29	20.02	255–355	Nevadesin (5,7-dihydroxy-6,8,4′-trimethoxyflavone)	C_18_H_15_O_7_^−^	343.08123	343.08063	−1.75	313.03363(di-demethylated molecule), 193.01280
30	20.21	254–330–354	Polymethoxylated flavonol (possibly casticin)	C_19_H_17_O_8_^−^	373.09103	373.09179	−2.03	358.06799 (demethylated molecule), 343.04413, (didemethylated molecule) 317.03029; 299.01791;177.01866
31	20.23	280	Unknown (possibly botryenalol)	C_17_H_25_O_4_^−^	293.17474	293.17380	2.51	279.1616 (demethylated molecule)

* Compounds detected using spiking experiments with authentic standards.

## Supplementary Materials

The following are available online at https://www.mdpi.com/1420-3049/24/6/1085/s1.
