# Supplementary material for: Antioxidant, Gastroprotective, Cytotoxic Activities and UHPLC PDA-Q Orbitrap Mass Spectrometry Identification of Metabolites in Baccharis grisebachii Decoction
Source: Molecules. 2019 Mar 19;24(6):1085. doi: 10.3390/molecules24061085 (PMC6472192; doi:10.3390/molecules24061085)
Supplement: Supplementary file 1 [file molecules-24-01085-s001.zip › supplementary/Bioethical approval document Gómez el al.,.pdf]

Universidad Nacional de San Luis
Facultad de Química Bioquímica y Farmacia
Secretaría de Ciencia y Técnica

ES COPIA
ALBA SUSANA OJEDA
Jefe Dpto. Despacho y M. Entrada
Fac. Qca. Bqca y Fcia
UNSL

SAN LUIS, 19 OCT 2017

VISTO:

El EXP-USL: 12406/2017 mediante el cual la Coordinadora del Comité Institucional de Cuidado y Uso de Animales (C.I.C.U.A.) eleva la nómina de Protocolos de Experimentación Aprobados durante el año 2017, y

CONSIDERANDO:

Que los Docentes Investigadores en cumplimiento de las Ordenanzas vigentes han presentado los Protocolos de Experimentación con Animales en tiempo y forma.-

Que el Comité Institucional de Cuidado y Uso de Animales (C.I.C.U.A) ha revisado, aprobado y elevado dichos Protocolos a la Facultad.-

Que se considera importante su protocolización ya que la mayoría de los Docentes-Investigadores necesitan contar con la misma para publicar en revistas de ámbito Nacional e Internacional.-

Que en la Sesión Ordinaria del 9 de Octubre del corriente año, el Consejo Directivo aprobó por unanimidad la lista de Protocolos de Experimentación con Animales.-

Por ello y en uso de sus atribuciones,

EL CONSEJO DIRECTIVO DE LA FACULTAD DE
QUIMICA, BIOQUÍMICA Y FARMACIA
RESUELVE:

ARTICULO 1°.- Aprobar la nómina de Protocolos de Experimentación realizados durante el año 2017 por Docentes- Investigadores de esta Facultad, según consta en el Anexo Único de la presente disposición.-

ARTICULO 2°.- Comuníquese, insértese en el Libro de Resoluciones de la Facultad publíquese en el Digesto Administrativo de la Universidad y archívese.-

RESOLUCION N° 296-17

ABL.

Dr. Sebastián Antonio Andujar
Secretario Ciencia y Técnica
Fac. Qca. Bqca. y Farmacia
U.N.S.L.

Dra. Mercedes Edith Campderros
Decana
Fac. Qca. Bqca. y Farmacia
U.N.S.L.

Universidad Nacional de San Luis
Facultad de Química Bioquímica y Farmacia
Secretaría de Ciencia y Técnica

ES COPIA
ALBA SUSANA OJEDA
Jefe Dpto. Despacho y M. Entrada
Fac. Qca. Bqca. y Fcia
UNSL

**B-282/17**	8701/2017	Caviedes vidal, Enrique	Fisiología ecológica de vertebrados	Caviedes vidal, Enrique	Efecto de la preferencia/selección de dieta sobre el sistema inmunológico en una especie granívora estricta, los diamantes mandarines (Aves, *Taeniopygia guttata*)
**F-283/17**	9557/2017	María, Alejandra	Evaluación farmacológica y toxicológica de productos naturales. Relación estructura-función	Garro, María	Evaluación del efecto curativo de *Lithraea molleoides* en úlcera gástrica en ratas
**F-284/17**	8837/2017	María, Alejandra	Evaluación farmacológica y toxicológica de productos naturales. Relación estructura-función	María, Alejandra	Actividad antiulcerosa del infuso de *Baccharis grisebachii* Hieron (Asteraceae)
**B-285/17**	9534/2017	Anzulovich Ana	Bases cronobiológicas del envejecimiento y patologias relacionadas	Cargnelutti , Ethel	Extracción de bazo para cultivo primario y puesta a punto de Western blot para determinar proteinas del reloj

Dra. [illegible] Farmacia
Fac. [illegible] U.N.S.L.

Sebastián Andujar
Secretario Ciencia y Técnica
Fac. Qca. Bqca. y Farmacia
U.N.S.L.

Corresp.Resol. Nº 296 17
