# Supplementary material for: Antioxidant, Gastroprotective, Cytotoxic Activities and UHPLC PDA-Q Orbitrap Mass Spectrometry Identification of Metabolites in Baccharis grisebachii Decoction
Source: Molecules. 2019 Mar 19;24(6):1085. doi: 10.3390/molecules24061085 (PMC6472192; doi:10.3390/molecules24061085)
Supplement: Supplementary file 1 [file molecules-24-01085-s001.zip › supplementary/Supplementary material for the article Gómez et al.,.pdf]

Supplementary material for the article: UHPLC-MS metabolome fingerprinting, anti-ulcer, antioxidant and cytotoxicity activities of *Baccharis grisebachii* lyophilized decoction.

Fig. S1. Full HR orbitrap MS spectra and tentative structures of some detected peaks (peaks 7, 8, 11, 15, 19, 21, 23, 24, 26, 29, and 30), signal at 197 is an artifact of the solvent system).

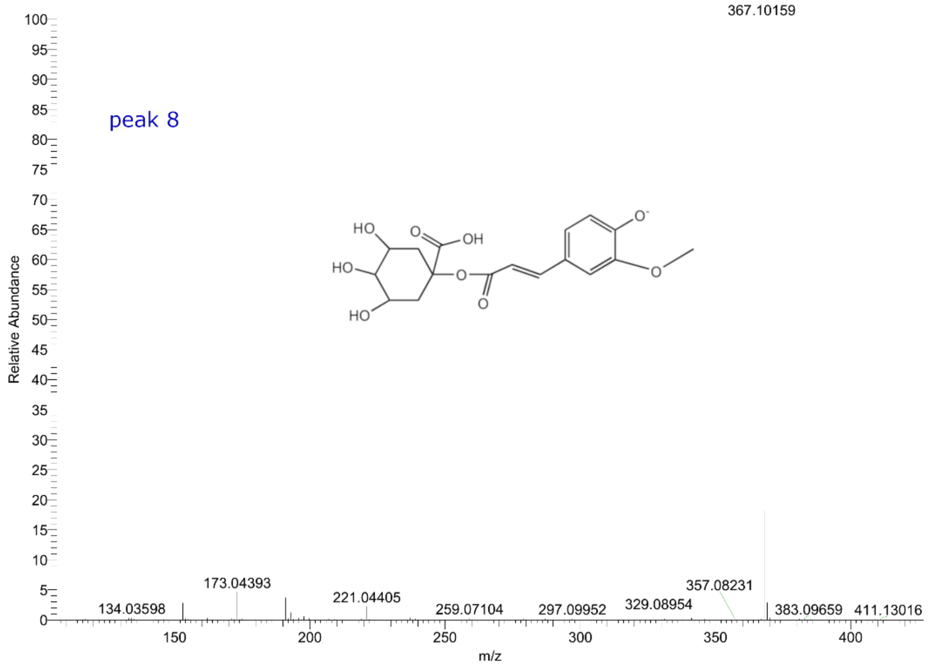

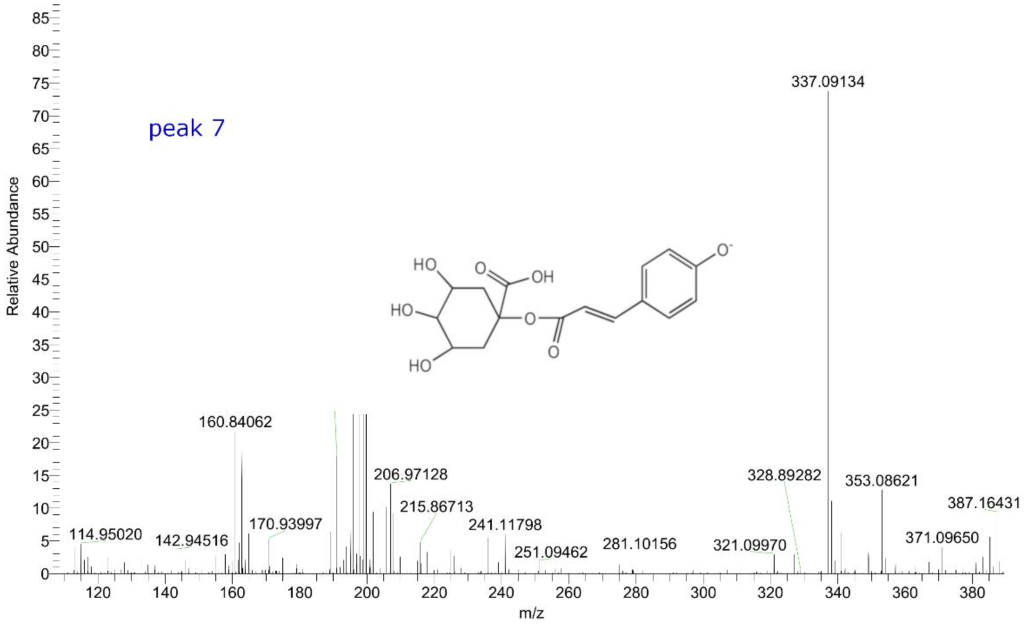

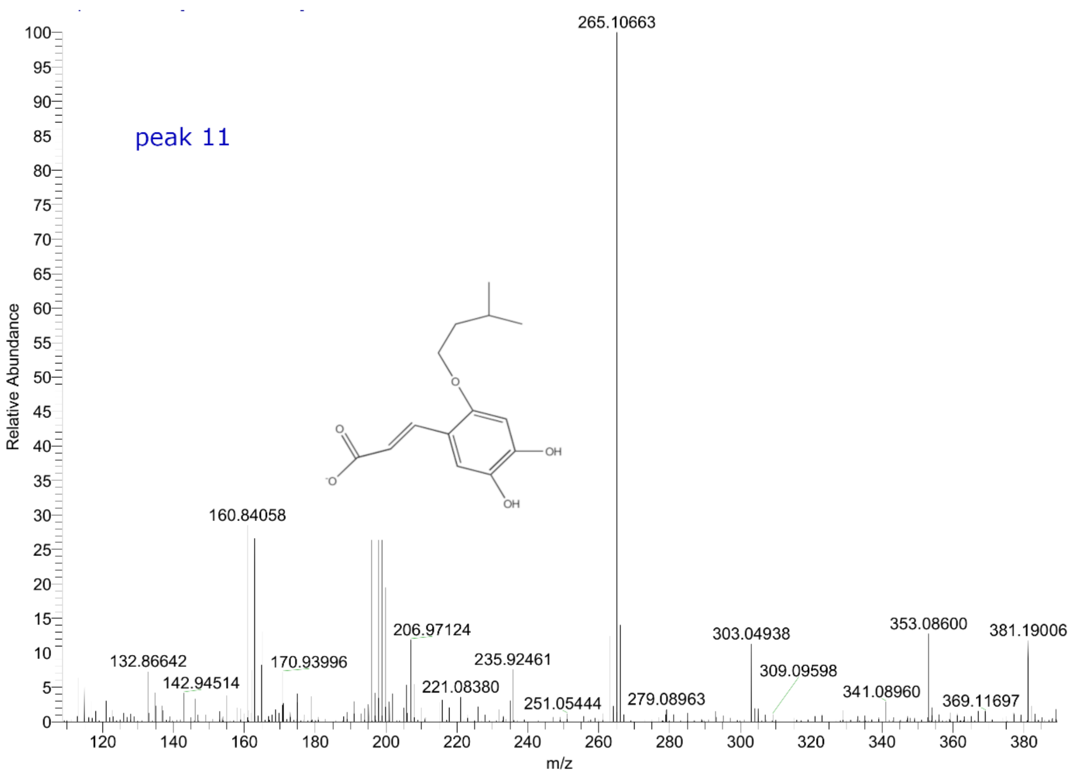
peak 11
265.10663
160.84058
206.97124
132.86642
170.93996
235.92461
142.94514
221.08380
251.05444
279.08963
303.04938
309.09598
353.08600
381.19006
341.08960
369.11697
Relative Abundance
m/z
OH
OH
O

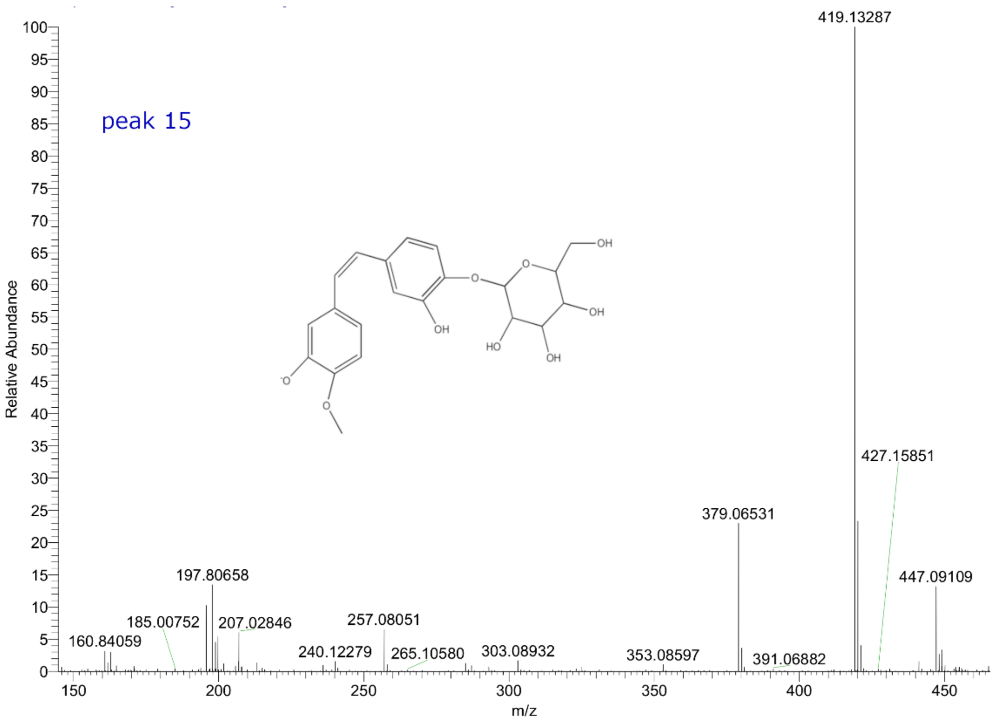
peak 15
419.13287
427.15851
379.06531
447.09109
197.80658
185.00752
207.02846
257.08051
160.84059
240.12279
265.10580
303.08932
353.08597
391.06882
Relative Abundance
m/z
OH
OH
OH
HO
OH
O

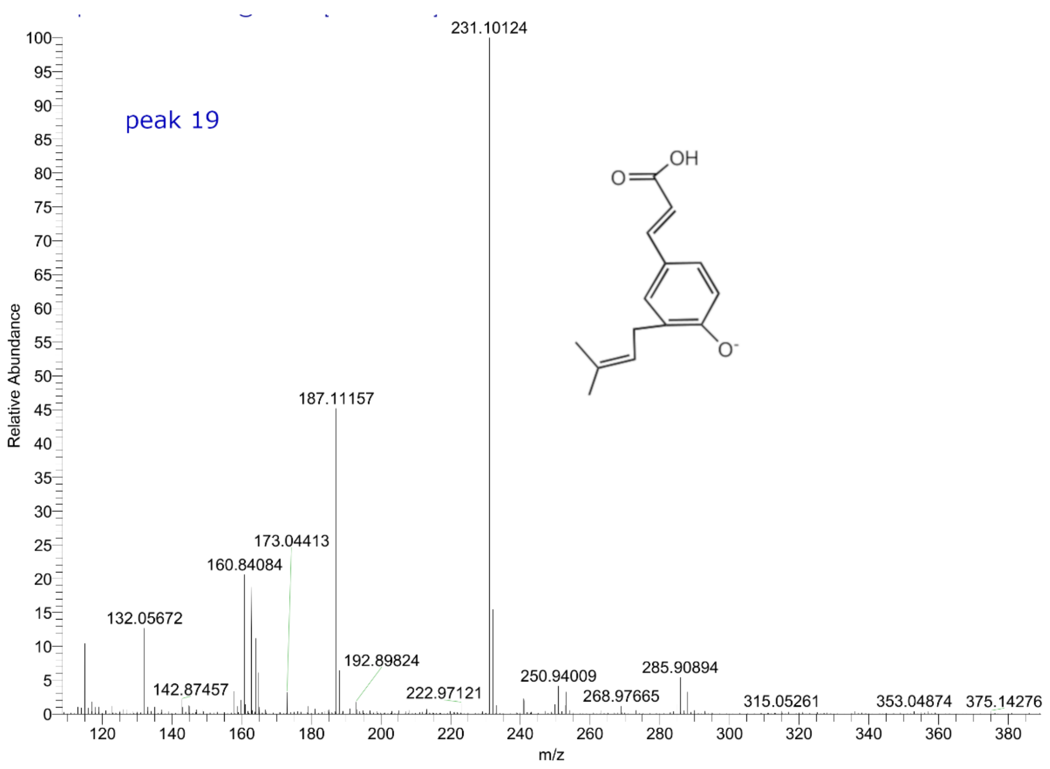
peak 19
Relative Abundance
m/z
231.10124
187.11157
173.04413
160.84084
132.05672
192.89824
142.87457
222.97121
250.94009
268.97665
285.90894
315.05261
353.04874
375.14276
OH
O
O-

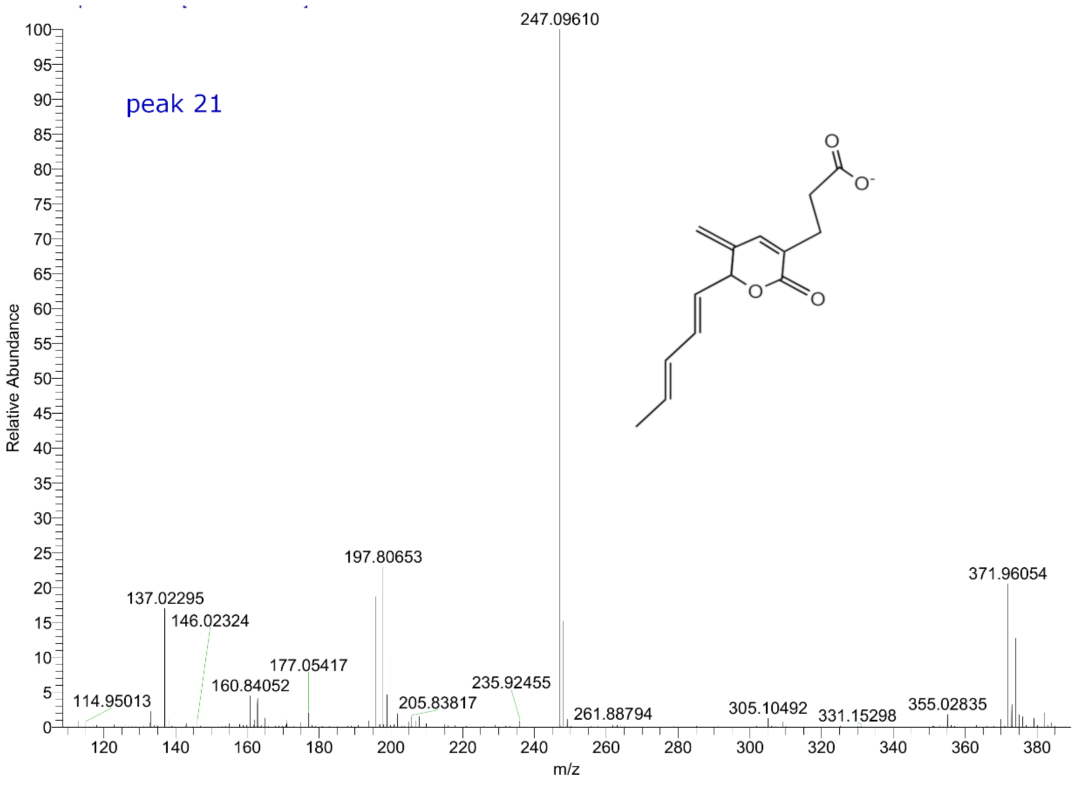
peak 21
Relative Abundance
m/z
247.09610
197.80653
137.02295
146.02324
177.05417
160.84052
114.95013
205.83817
235.92455
261.88794
305.10492
331.15298
355.02835
371.96054
O
O-
O
O

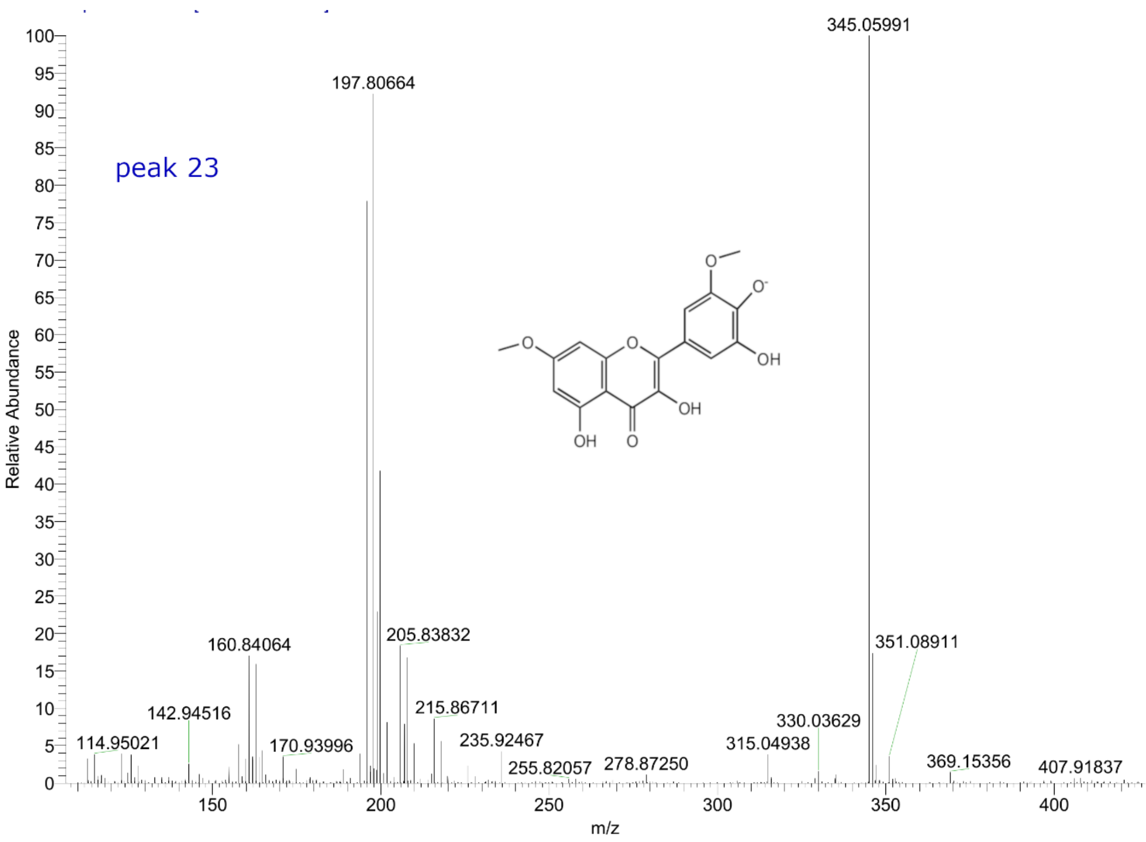
peak 23
345.05991
197.80664
160.84064
205.83832
351.08911
142.94516
215.86711
330.03629
114.95021
170.93996
235.92467
315.04938
255.82057
278.87250
369.15356
407.91837
Relative Abundance
m/z
OH
O

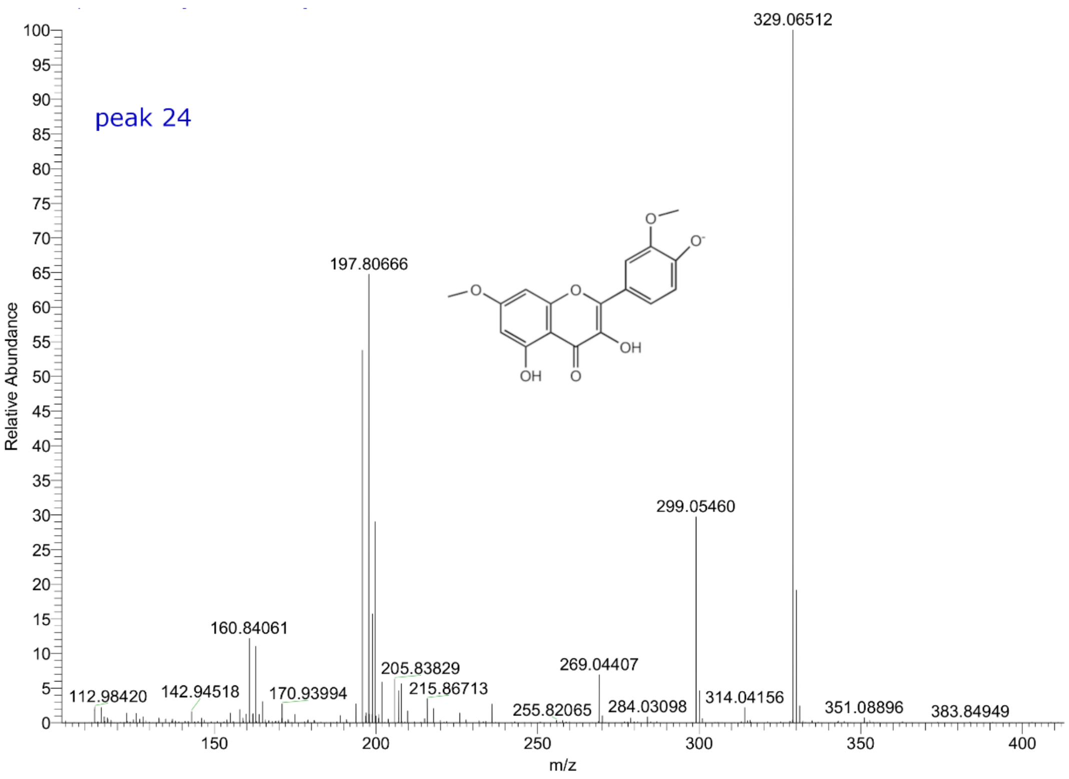
peak 24
329.06512
197.80666
299.05460
160.84061
269.04407
205.83829
215.86713
112.98420
142.94518
170.93994
255.82065
284.03098
314.04156
351.08896
383.84949
Relative Abundance
m/z
OH
O

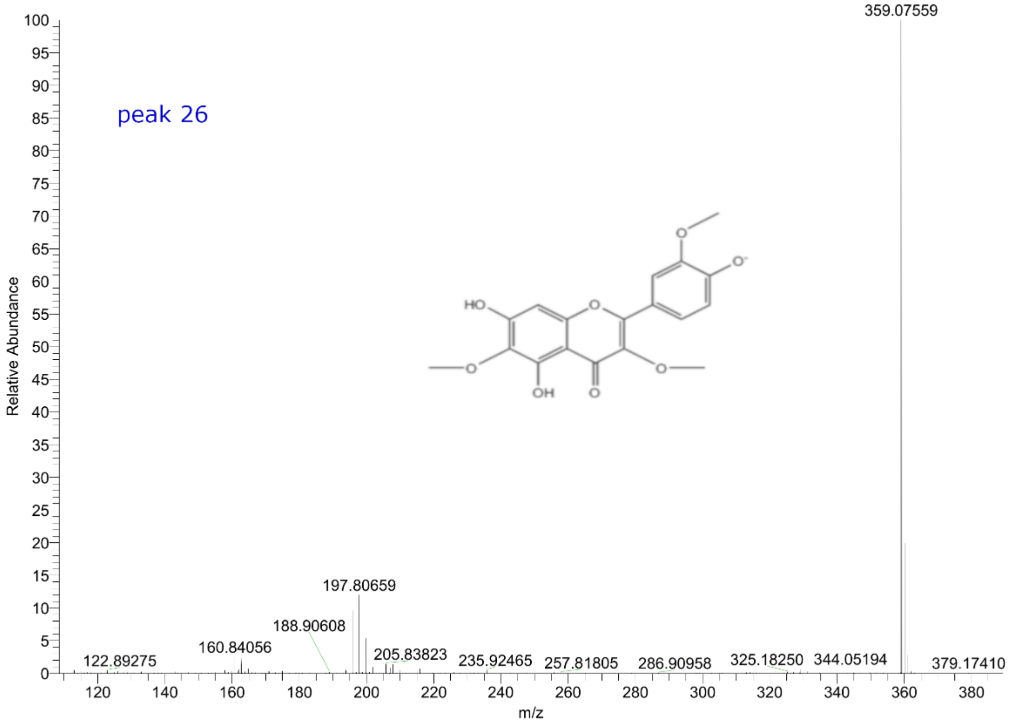
359.07559
peak 26
Relative Abundance
m/z
197.80659
188.90608
160.84056
122.89275
205.83823
235.92465
257.81805
286.90958
325.18250
344.05194
379.17410
HO
OH
O

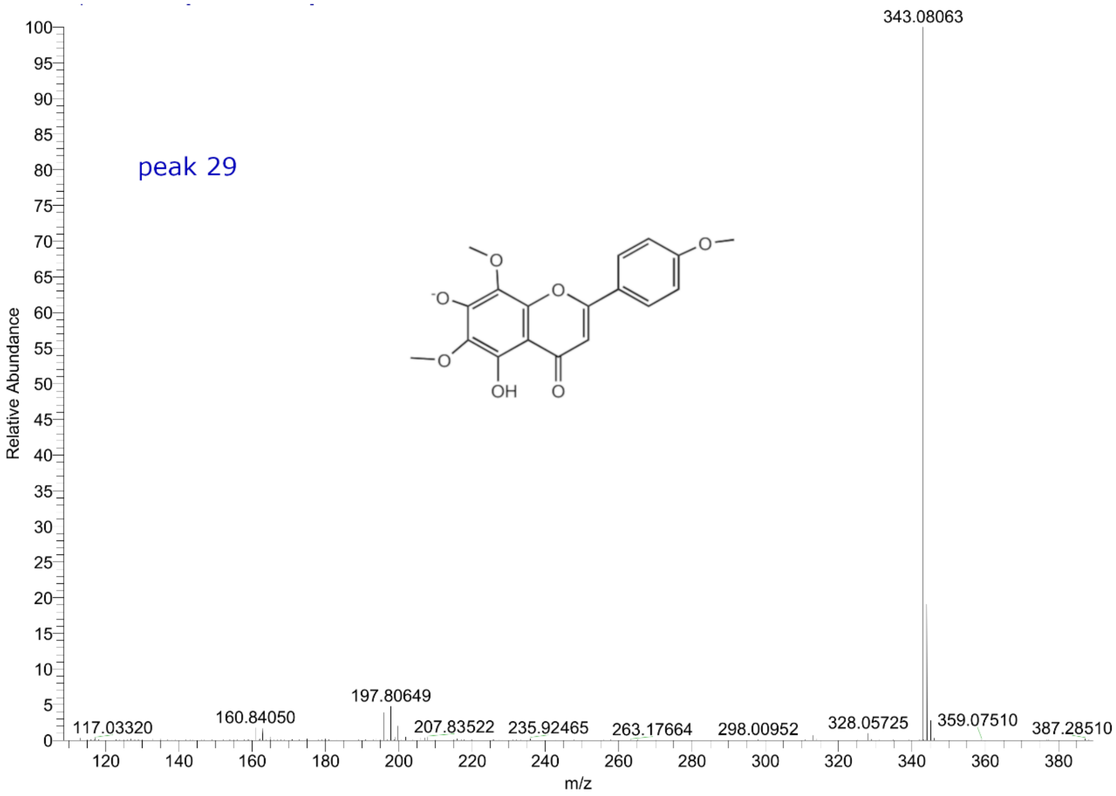
343.08063
peak 29
Relative Abundance
m/z
197.80649
160.84050
117.03320
207.83522
235.92465
263.17664
298.00952
328.05725
359.07510
387.28510
OH
O

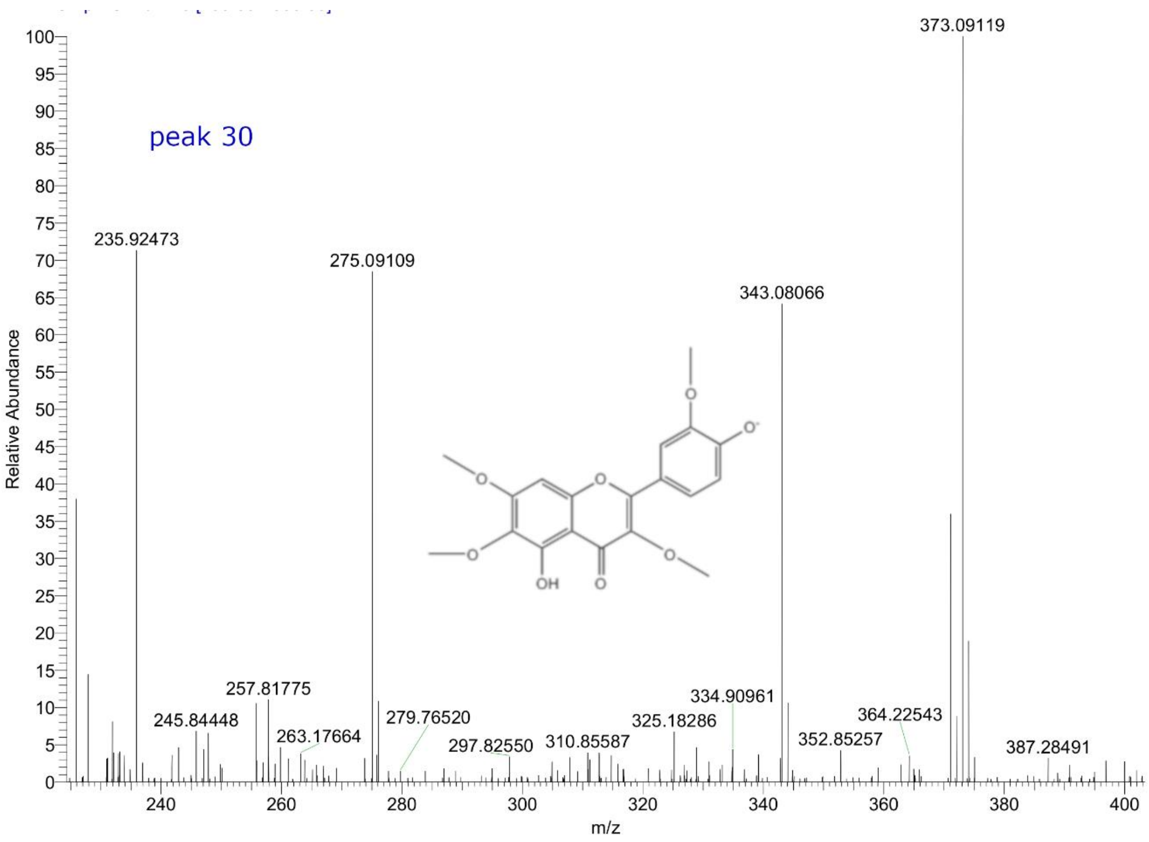

Mass spectrometry conditions: The HESI parameters were as follows: sheath gas flow rate, 75 units; auxiliary gas unit flow rate, 20; capillary temperature, 400 °C; auxiliary gas heater temperature, 500 °C; spray voltage, 2500 V (for ESI-); and S lens, RF level 30. Full scan data in positive and negative (it was chosen the negative for phenolic compounds) were acquired at a resolving power of 70,000 FWHM (full width half maximum) at m/z 200. For the compounds of interest, a scan range of *m*/*z* 100-1000 was chosen; the automatic gain control (AGC) was set at 3×10^6 and the injection time was set to 200 ms. The scan-rate was set at 2 scans s^{-1}. External calibration was performed using a calibration solution in positive and negative modes. For confirmation purposes, a targeted MS-MS analysis was performed using the mass inclusion list, with a 30 s time window, with the Orbitrap spectrometer operating both in positive and negative modes at 17,500 FWHM (m/z 200). The AGC target was set to 2×10^5, with the maximum injection time of 20 ms. The precursor ions were filtered by the quadrupole, which operated at an isolation window of *m*/*z* 2. The fore vacuum, high vacuum and ultrahigh vacuum were maintained at approximately 2 mbar, from 105 and below 1010 mbar, respectively. Collision energy (HCD cell) was operated at 5 ev while full MS detections at energy: 0 ev for phenolic compound. Detection was based on calculated exact mass and on retention time of all compounds. The mass tolerance window was set to 5 ppm for the two modes.
